# Pro Re Nata Use of Psychotropic Medications in Assisted Living

**DOI:** 10.1093/geroni/igaa057.2482

**Published:** 2020-12-16

**Authors:** Paula Carder, Sheryl Zimmerman, Christopher Wretman, Sarah Dys, Philip Sloane

**Affiliations:** 1 Portland State University, Portland, Oregon, United States; 2 University of North Carolina at Chapel Hill, Chapel Hill, North Carolina, United States; 3 Institute on Aging, Portland, Oregon, United States

## Abstract

This study examined the use of pro re nata (PRN, or as needed) psychotropic medications among assisted living (AL) residents. We examined prescriptions and administrations, and compared use based on dementia diagnosis. Data sources included interviews with administrators of 250 AL communities in 7 states and medication administration record review for the prior 7 days; analyses were weighted to the entire state. The percent of all residents prescribed a PRN psychotropic medication was 10.3%. However, residents with a dementia diagnosis were twice as likely to have a PRN psychotropic prescription (15.2% versus 7.2%; p<.001). The majority of psychotropic medications prescribed and administered were for anxiolytics/hypnotics rather than antipsychotics. Additional resident-level factors significantly associated with higher PRN prescribing included psychiatric diagnosis, incontinence, hospice use, confusion/disorientation, and agitation. We summarize these and other findings in the context of state regulatory requirements for staffing, chemical restraints, and dementia care.

